# Metformin Ameliorates Dysfunctional Traits of Glibenclamide- and Glucose-Induced Insulin Secretion by Suppression of Imposed Overactivity of the Islet Nitric Oxide Synthase-NO System

**DOI:** 10.1371/journal.pone.0165668

**Published:** 2016-11-07

**Authors:** Ingmar Lundquist, Israa Mohammed Al-Amily, Sandra Meidute Abaraviciene, Albert Salehi

**Affiliations:** 1 Department of Clinical Science, SUS, Division of Islet Cell Physiology University of Lund, Malmö, Sweden; 2 Department of Experimental Medical Science, University of Lund, Lund, Sweden; 3 Dept. of Physiology, Biochemistry, Microbiology and Laboratory Medicine Vilnius University, and Dep. of Regenerative Medicine, State Research Institute Center for Innovative Medicine, Vilnius, Lithuania; 4 Department of Neuroscience and Physiology, Metabolic Research Unit, University of Gothenburg, Gothenburg, Sweden; Consiglio Nazionale delle Ricerche, ITALY

## Abstract

Metformin lowers diabetic blood glucose primarily by reducing hepatic gluconeogenesis and increasing peripheral glucose uptake. However, possible effects by metformin on beta-cell function are incompletely understood. We speculated that metformin might positively influence insulin secretion through impacting the beta-cell nitric oxide synthase (NOS)-NO system, a negative modulator of glucose-stimulated insulin release. In short-time incubations with isolated murine islets either glibenclamide or high glucose augmented insulin release associated with increased NO production from both neural and inducible NOS. Metformin addition suppressed the augmented NO generation coinciding with amplified insulin release. Islet culturing with glibenclamide or high glucose revealed pronounced fluorescence of inducible NOS in the beta-cells being abolished by metformin co-culturing. These findings were reflected in medium nitrite-nitrate levels. A glucose challenge following islet culturing with glibenclamide or high glucose revealed markedly impaired insulin response. Metformin co-culturing restored this response. Culturing murine islets and human islets from controls and type 2 diabetics with high glucose or high glucose + glibenclamide induced a pronounced decrease of cell viability being remarkably restored by metformin co-culturing. We show here, that imposed overactivity of the beta-cell NOS-NO system by glibenclamide or high glucose leads to insulin secretory dysfunction and reduced cell viability and also, importantly, that these effects are relieved by metformin inhibiting beta-cell NO overproduction from both neural and inducible NOS thus ameliorating a concealed negative influence by NO induced by sulfonylurea treatment and/or high glucose levels. This double-edged effect of glibenclamide on the beta-cellsuggests sulfonylurea monotherapy in type 2 diabetes being avoided.

## Introduction

The biguanide metformin is presently recommended as a first-line drug in the treatment of impaired glucose tolerance and manifest type 2 diabetes [1]. It is well known that metformin has important extrapancreatic actions in lowering blood glucose by enhancing peripheral glucose uptake and reducing hepatic gluconeogenesis [2]. However, metformin has also been suggested to display a positive action on the pancreatic beta-cell. Thus it has been reported that metformin may modestly augment glucose-stimulated insulin release in isolated human islets [3,4]. The mechanisms underlying this effect on the pancreatic beta-cell are presently still unclear. In current clinical praxis, when treating type 2 diabetes, metformin is often used together with sulfonylurea drugs such as *e*.*g*. glibenclamide. Although sulfonylureas also have efficient glucose-lowering properties, such a treatment has evolved, because clinical studies have revealed that sulfonylurea monotherapy has a pronounced tendency to fail over time [5]. Hence it seemed of great importance to elucidate what unexplained mechanisms could underlie the less understood effects of metformin on beta-cell function both after exposure to sulfonylurea treatment and to elevated glucose levels.

In previous studies we have emphasized the importance of islet generation of nitric oxide (NO) for the function and viability of the beta-cell [6–9]. The neuronal constitutive NO-synthase (ncNOS, also named nNOS or NOS1) is constitutively expressed in the beta-cells of both rodent and human islets[10–12], while the inducible NOS (iNOS, also named NOS2) is expressed mainly at high persisting glucose levels and/or after cytokine impact on the beta-cell [9,13–15]. We have shown in normal rodent islets that ncNOS-derived NO is immediately released upon glucose stimulation both *in vivo* and *in vitro* [6,7], while iNOS-derived NO is apparent first at the latter part of a 60 min exposure to an approximate glucose concentration of ≥9–10 mmol/l [7]. Moreover, ncNOS protein is in great part associated with the insulin secretory granules and mitochondria [11,12], whereas iNOS protein is more uniformly distributed in the beta-cell cytoplasm [16]. Thus beta-cell ncNOS is conveniently located to immediately impact and regulate the secretory machinery after acute glucose stimulation, while iNOS-derived NO has been shown to be insufficient in this context [17] and more likely contributes to β-cell dysfunction during persisting hyperglycemia and/or hyperlipidemia and the development of nonimmunogenic type 2 diabetes over time [8,9,15,18–23]. In view of these findings we found it mandatory to study the possible influence and pattern of NO generation from both ncNOS and iNOS activities in relation to insulin release from isolated murine islets following incubation with glibenclamide and/or high glucose in the presence or absence of metformin.

Our results strongly suggest that imposed overactivity of the islet NOS-NO system induced by glibenclamide and/or high glucose could markedly contribute to a progressive dysfunction of the insulin secretory mechanisms being counteracted by a beneficial action of metformin, an effect which in large part is due to a strong suppressive effect by the biguanide on the upregulated activity of the NOS-NO system in the beta-cell.

## Materials and Methods

### Animals

Female mice of the Naval Medical Research Institute (NMRI) strain (B&K, Sollentuna, Sweden), weighing 25–35 g, were used throughout the experiments. They were given a standard pellet diet (B&K) and tap water ad libitum. The experimental procedures were approved by the Ethics Committee for Animal Research at University of Lund, Sweden.

### Drugs and chemicals

Glibenclamide (also named glyburide) was obtained from ICN Biochemicals Inc (USA), Metformin and collagenase were from Sigma Aldrich (USA). All other drugs and chemicals were from British Drug Houses Ltd, Poole, UK or Merck AG, Darmstadt, Germany. The radioimmunoassay kits for insulin determinations were obtained from Millipore (Stockholm, Sweden).

### In vitro insulin release studies

Preparation of isolated pancreatic islets from the mouse was performed by retrograde injection of a collagenase solution via the bile-pancreatic duct [24]. In batch incubation experiments, freshly isolated islets were preincubated for 30 min at 37°C in Krebs–Ringer bicarbonate buffer, pH 7.4, supplemented with 10 mmol/l HEPES, 1 mmol /1 glucose and 0.1% bovine serum albumin [25]. Each incubation vial was gassed with 95% O_2_ and 5% CO_2_ to obtain constant pH and oxygenation. After preincubation, the buffer was changed to a fresh KRB buffer supplemented with the different test agents, and the islets (12 islets per 1.0 ml of medium in each incubation vial) were incubated for 60 min. All incubations were performed at 37° C in an incubation box (30 cycles per min). Immediately after incubation aliquots of the medium were removed and frozen for subsequent assay of insulin [25]. In culture experiments, the murine islets were cultured for 24 h in RPMI-1640 (SVA, Uppsala, Sweden) containing 7 mmol/l glucose supplemented with 10% calf serum, 100 U/ml penicillin and 10 μg/ml streptomycin + added glucose and drugs as described. After culture and washing, the islets were preincubated and incubated as described above.

### Assay of islet NOS activities

Preincubation and incubation of freshly isolated islets were performed as stated above with the exception that each incubation vial contained 250 islets in 1.5 ml of buffer solution [7]. After an incubation period of 60 min, aliquots of the medium were removed for determination of insulin. Thereafter, the islets were washed and collected in 840 μl of buffer, containing 20 mmol/l HEPES, 0.5 mmol/l EDTA and 1.0 mmol/l D,L-dithiothreitol and stored at -20 C. On the day of the assay, the islets were sonicated on ice and for measuring ncNOS activity the buffer solution was enriched with 0.45 mmol/l CaCl_2_, 2 mmol/l NADPH, 25 U/ml calmodulin and 0.2 mmol/l L-arginine. For the determination of iNOS activity, both Ca^2+^ and calmodulin were omitted from the buffer [7,25]. The homogenate solution was then incubated at 37° C under constant air bubbling, 1.0 ml/min for 2 h. Aliquots of the incubated homogenate (200 μl) were then passed through an 1-ml Amprep CBA cation exchange column for determination of L-citrulline by high-performance liquid chromatography (HPLC). The method has been described in detail [7]. As L-citrulline and NO are generated in equimolar amounts, and as L-citrulline is stable, whereas NO is not, L-citrulline is the preferred parameter when measuring NO production. Protein was determined according to Bradford [26] on samples from the original homogenate.

### Immunofluorescence and confocal microscopy

After 24 h culturing, the islets were washed (three times) and fixed with 4% formaldehyde, permeabilized with 5% Triton X-100 and unspecific sites were blocked with 5% Normal Donkey Serum (Jackson Immunoresearch Laboratories Inc., West Grove, PA, USA). Polyclonal anti-iNOS antibody (StressGen Biotechnologies Corp., Victoria, BC, Canada) (1:100) in combination with Cy2-conjugated anti-rabbit IgG (Jackson Immunoresearch Laboratories Inc.) (1:150) was used to detect iNOS. For staining of insulin, islets were incubated with a guinea pig-raised anti-insulin antibody (Eurodiagnostica, Malmö, Sweden) (1:1000) followed by incubation with a Cy5-conjugated anti-guinea pig IgG antibody (Jackson Immunoresearch Laboratories Inc.) (1:150). The fluorescence was visualized with a Zeiss LSM510 confocal microscope (Carl Zeiss Inc., Jena, Germany) by sequentially scanning at (excitation/emission) 488/ 505–530 nm (Cy2) and 633/>650 nm (Cy5). For scoring of iNOS-positive cells in islet tissue, multiple fields for each section were analyzed under blind conditions. The mean fluorescent intensity of cellular iNOS was analyzed using Zeiss LSM5 analysis software. After co-localization of iNOS and insulin, iNOS fluorescence was quantified pixel by pixel using Zen 2009 (Carl Zeiss, Oberkochen, Germany) software. All fluorescence intensity measurements were performed with randomly selected islets from 6–8 mice in each group.

### Determination of nitrite and nitrate

The released nitrite and nitrate in the culture medium were determined using a commercially available Colorimetric Assay Kit following the manufacturer’s instruction (Cayman Chemical Company, Ann Arbor, MI, USA).

### Viability tests with isolated murine and human islets

Because of limited availability of human islets, such islets could only be used in the viability test. After 72 h culture of murine or human islets with basal or 20 mmol/l glucose in the absence or presence of different test agents the islets were dispersed into single cells using Ca^2+^-free medium. Measurement of cell viability was performed using MTS reagent according to manufacturer’s instructions (Promega, USA). The isolated human islets were provided by Nordic Network for Clinical Islet Transplantation (Olle Korsgren, Uppsala University, Sweden) [9]. Donor characteristics are summarized in Table 1.

**Table 1:** Characteristics of non-diabetic (ND) and type-2 diabetic (T2D) organ donors. The sex, age, BMI and HbA1c are shown. Pancreatic islets from donors with HbA1c <6.2% were considered non-diabetic (ND) and islets from donors with HbA1c>6.2% or history of diabetes were considered as diabetic islets in our study. ***p<0.001 for ND vs T2D.

Gender	Age	BMI	HbA1c
ND: 6 male and 2 female	51.4±5.1	26.9±1.2	5.4±0.13
T2D: 1 male and 3 female	63.2±2.8	30.4±0.8	6.4±0.18***

### Statistics

The statistical differences between groups were determined by an analysis of variance followed by Tukey-Kramer’s multiple comparisons test or Student’s t-test where applicable.

## Results

### Effects of metformin and glibenclamide on islet activities of ncNOS and iNOS in relation to insulin release at a basal glucose concentration of 7 mmol/l

**Fig 1:**
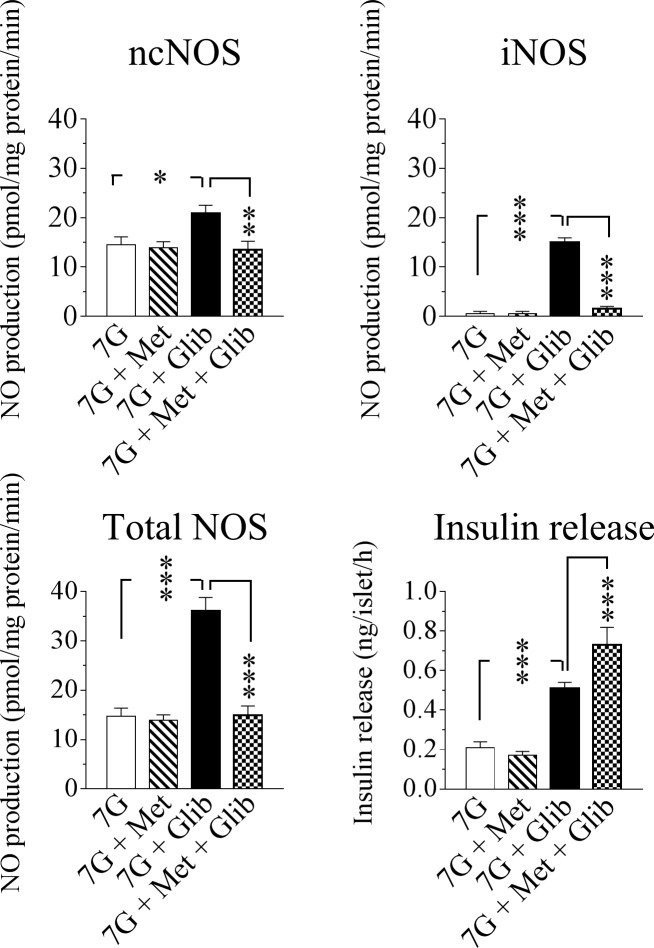
Effect of metformin on the NOS-NO system and glibenclamide-induced insulin release. ncNOS, iNOS and total NOS activities as well as insulin release in murine islets incubated at 7 mmol/l glucose (7G) for 60 min in the absence and presence of 20 μmol/l metformin (Met) or 5 μmol/l glibenclamide (Glib) or both. Means ± SEM for 6–9 batches of islets in each group are shown. *p<0.05; **p<0.01; ***p<0.001

To elucidate whether glibenclamide and/or metformin would affect the islet activities of both ncNOS and iNOS during a short-time incubation we performed a series of experiments with basal 7 mmol/l glucose (7G), 7G + metformin, 7G + glibenclamide and 7G + metformin + glibenclamide (Fig 1). While metformin did not have any appreciable impact on islet ncNOS or iNOS activities at this basal glucose level, glibenclamide had a marked stimulatory effect on the production of both ncNOS-derived NO and in a particular iNOS-derived NO (Fig 1). Moreover and notably, metformin totally suppressed glibenclamide-stimulated NO generation emanating from both ncNOS and iNOS back to control levels and in addition induced a marked augmentation of glibenclamide-induced insulin release (Fig 1).

### Effect of metformin on islet activities of ncNOS and iNOS in relation to insulin release at high glucose

**Fig 2:**
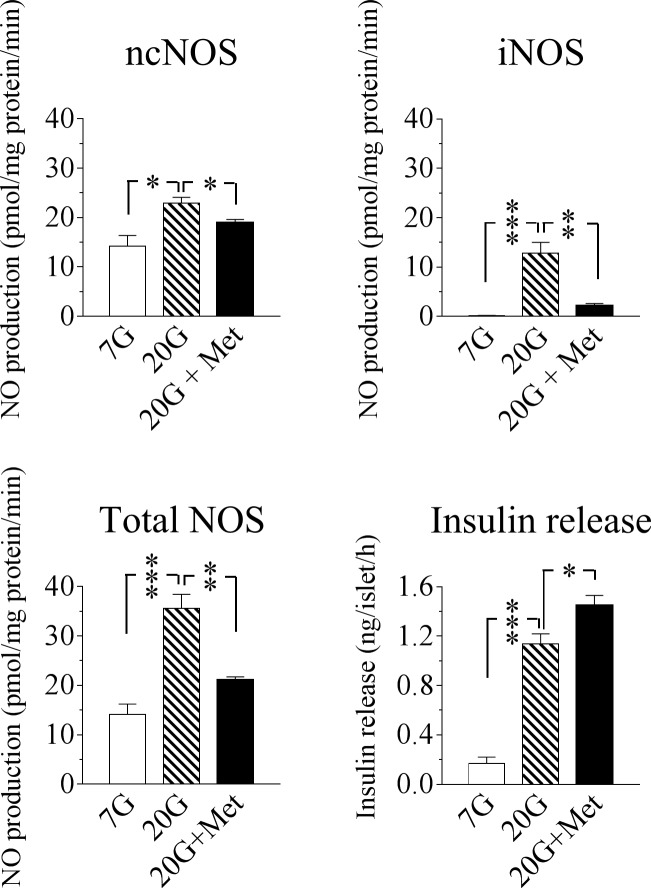
Effect of metformin on the NOS-NO system and glucose-induced insulin release. ncNOS, iNOS and total NOS activities as well as insulin release in murine islets incubated at 7 or 20 mmol/l glucose (7G or 20G) for 60 min in the absence and presence of 20 μmol/l metformin (Met). Means ± SEM for 5–6 batches of islets in each group are shown. *p<0.05; **p<0.01; ***p<0.001

Recent studies have shown that metformin might have a modest stimulatory effect on glucose-induced insulin release in isolated human islets, although the underlying mechanisms are unclear [3,4]. Fig 2 shows that high glucose (20 mmol/l) induced an increase in the activity of both ncNOS and iNOS in our murine islets. In line with our hypothesis we tested whether metformin would have any impact on the activities of ncNOS and iNOS at this glucose level in short-time incubations. As shown in Fig 2 metformin induced a modest inhibition of ncNOS activity being associated with a significant augmentation of glucose-stimulated insulin release. Moreover, metformin induced an almost total suppression of iNOS-derived NO. Thus total NOS activity was suppressed back almost to the control level by metformin addition (Fig 2).

### Islet culturing with glibenclamide or high glucose in the absence or presence of metformin

The more long-time influence of metformin on islet function in relation to the NOS-NO system was studied following islet culture for 24h at basal (7 mmol/l) glucose ± glibenclamide and/or metformin as well as at high glucose (20 mmol/l) ± metformin.

**Fig 3:**
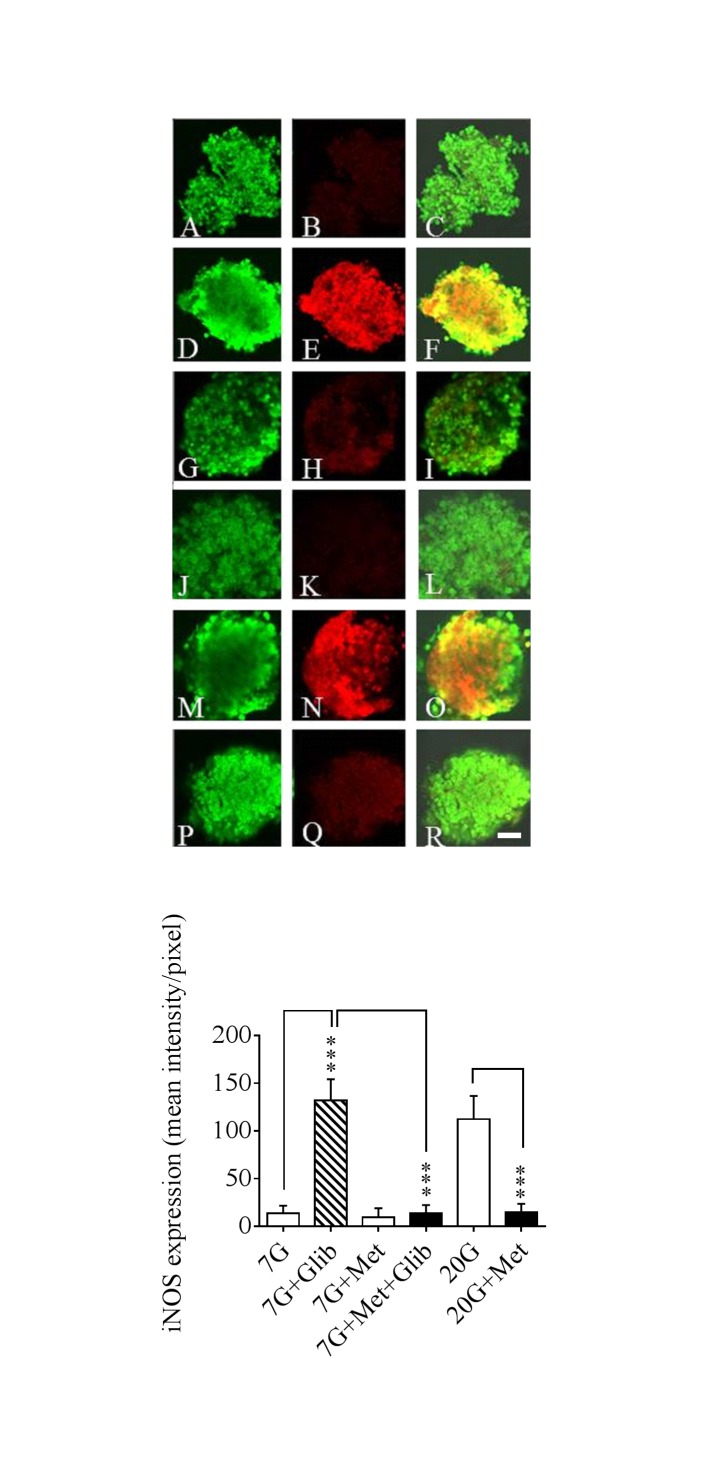
Confocal microscopy and fluorescence intensity after 24 h culturing. Confocal microscopy and fluorescence intensity measurements of murine islets cultured for 24h at 7 mmol/l glucose (7G) ± 5 μmol/l glibenclamide (Glib) or 20 μmol/l metformin (Met) or both as well as at 20 mmol/l glucose (20G) ± 20 μmol/l metformin (Met). The islets were double-immunolabelled for insulin (A, D, G, J, M, P) and iNOS protein (B, E, H, K, N, Q). Insulin staining appears as green and iNOS as red staining, respectively. Co-localization of insulin/iNOS is seen as orange-yellowish fluorescence (C, F, I, L, O, R). Bars indicate 20 μm Lower part of the figure shows fluorescence intensity measurements quantified pixel by pixel using Zen 2009 software. Means ± SEM are shown for 19–34 observations in each group. ***p<0.001

**Fig 4:**
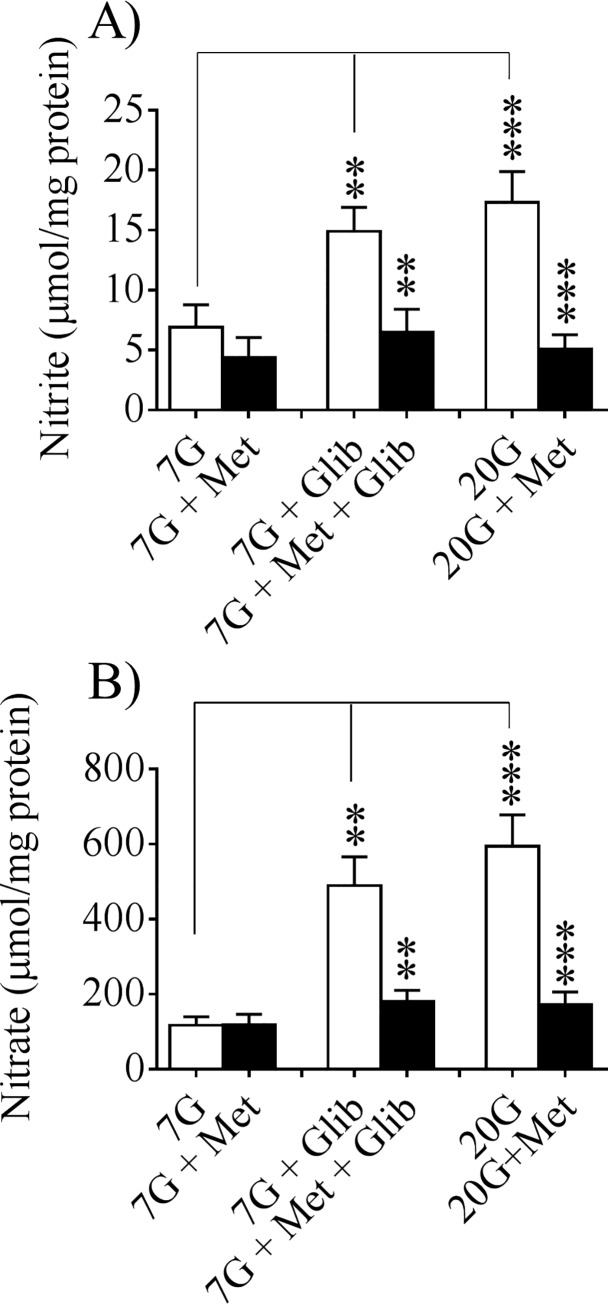
Medium nitrite and nitrate concentrations after 24 h culturing. Detection of nitrite (A) and nitrate (B) production from isolated murine islets cultured for 24 h with the different treatments as described on Fig 3. Means ± SEM for 6–8 batches of islets in each group are shown. **p<0.01; ***p<0.001

The culture period was thereafter followed by a glucose challenge to test the insulin secretory capacity after the different treatments. Fig 3 upper panel shows the distribution of iNOS protein in the beta-cells of the different treatment categories as revealed by confocal microscopy. Glibenclamide at basal glucose as well as high glucose by itself induced a marked expression of iNOS protein co-existing with insulin in the beta-cells. In the presence of metformin the iNOS fluorescence was totally abolished showing that metformin was able to powerfully counteract the induction of iNOS protein expression in beta-cells treated with glibenclamide or high glucose. Quantification by fluorescence intensity measurements (Fig 3 lower panel) confirmed the visual impression. Moreover, the production of nitrite (Fig 4A) and nitrate (Fig 4B) into the culture medium revealed a high activity of the NOS-NO system both at glibenclamide + 7 mmol/l glucose as well as at 20 mmol/l glucose. This overproduction of nitrite-nitrate was abolished by metformin (Fig 4A and 4B) thus showing a similar pattern as the confocal data.

**Fig 5:**
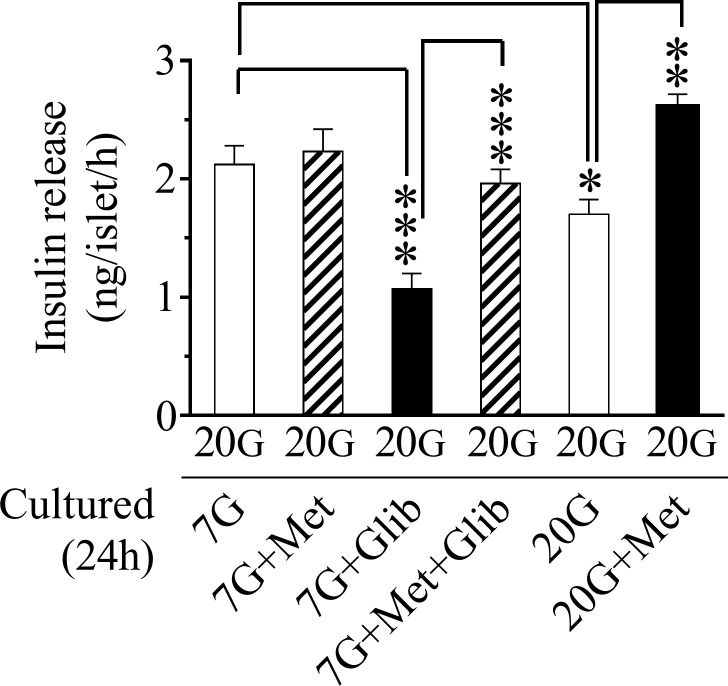
Insulin release following a glucose challenge after 24 h culturing. Isolated murine islets were cultured for 24 h at 7 mmol/l glucose (7G) in the absence or presence of 20 μmol/l metformin (Met) or 5 μmol/l glibenclamide (Glib) or both as well as at 20 mmol/l glucose (20G) ± 20 μmol/l metformin (Met) as denoted on the figure. Insulin release was then measured following a 20 mmol/l glucose challenge during a 60 min short-time incubation. There were 6–12 batches of islets in each group. Means ± SEM are shown. *p<0.05; **p<0.01; ***p<0.001

Finally, following the culture period, the islets from the different categories were washed, preincubated for 30 min, and then incubated for 60 min with high glucose (20 mmol/l) to test the insulin secretory capacity following the various treatments. Fig 5 shows that islets cultured with glibenclamide displayed a marked suppression of glucose-stimulated insulin release. Similarly, islets cultured at high glucose (20 mmol/l) showed a reduced insulin response to a subsequent glucose challenge. In contrast, islets cultured with glibenclamide + metformin or high glucose + metformin showed that the insulin response now had returned to normal levels, thus revealing a beneficial effect by metformin against the overactivity of the NOS-NO system.

### Metformin restores the reduced viability of murine islets and human non-diabetic and type 2 diabetic islets after culturing with high glucose or high glucose + glibenclamide

**Fig 6:**
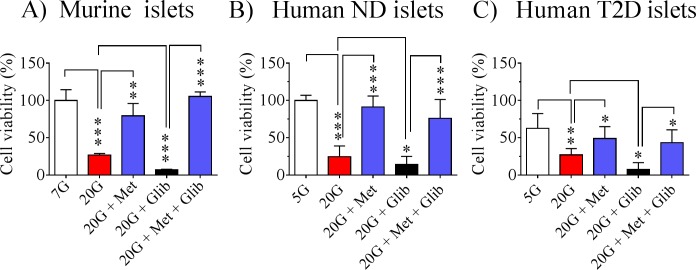
Cell viability after 72 h culturing. Effects on cell viability after treatment with metformin 20 μmol/l (Met), glibenclamide 5 μmol/l (Glib) or both on isolated murine islets (A) or isolated human islets from control donors (non-diabetic islets)(B) or type 2 diabetic donors (T2D islets)(C) cultured for 72 h at 20 mmol/l glucose. Control islets cultured at basal glucose are included. Means ± SEM for 4 observations (murine islets) or for 3–10 observations (human islets) are shown. *p<0.05; **p<0.01; ***p<0.001

To test whether metformin might have any direct impact on beta-cell viability (measuring mitochondrial reductive capacity) after treatment with either high glucose or glibenclamide + high glucose, we cultured murine islets as well as human non-diabetic and type 2 diabetic islets at basal glucose or 20 mmol/l glucose or at 20 mmol/l glucose + glibenclamide (Fig 6). There was a marked reduction in islet cell viability at 20 mmol/l glucose compared with culturing at basal glucose in both murine and human islets. This reduced viability was completely restored to normal by metformin (Fig 6A and 6B). Moreover, addition of glibenclamide together with glucose (20 mmol/l) reduced the cell viability to almost zero. Thus glibenclamide accentuated the decrease in viability induced by high glucose. Also this strong suppression of viability could be restored by metformin (Fig 6A and 6B). These effects were evident and quantitatively similar with both murine and human islets. Finally, we performed a similar viability test with islets isolated from type 2 diabetic donors (Fig 6C). These islets displayed the same pattern as normal human islets after the different treatments although, as expected, the initial control level at basal glucose was reduced (by approximately 40%) compared to normal non-diabetic human islets. However, the beneficial effect of metformin was clearly evident (Fig 6C).

## Discussion

It was shown several years ago that iNOS-derived NO, primarily produced from macrophages and invasive cells, with the resulting formation of reactive nitrogen species (RNS) and reactive oxygen species (ROS), has an important impact on the immunogenic and inflammatory destruction of islet beta-cells in type 1 diabetes [13–15]. In addition overactivity of the NOS-NO system within the beta-cells has been suggested to be implicated in the pathogenesis of nonimmunogenic nonobese type 2 diabetes in the young GK rat [18,19] as well as in human nonobese type 2 diabetes [9]. As previously mentioned we have found, in isolated islets from healthy mice and rats, that iNOS-derived NO appears already at the end of an incubation period of 60 min after exposure to high glucose[7]. ncNOS-derived NO, on the other hand, appears immediately and coincident with the glucose-stimulated insulin release as we have observed in perifused rat islets as well as *in vivo* and *in vitro* in mice [6,7]. Moreover, we have repeatedly found that this acutely ncNOS-derived NO exerts an inhibitory effect, a physiological negative feedback, on the insulin secretory process induced by glucose [6,7,16,19]. Hence, in the present study and for comparative reasons, we started to use an incubation time of 60 min in order to possibly detect both ncNOS as well as iNOS activities in relation to insulin secretion induced by glibenclamide, high glucose and/or metformin. We found here for the first time that glibenclamide, in the presence of basal glucose, had the ability to stimulate both ncNOS and iNOS activities to the same levels as observed in a comparative experiment with high glucose (20 mmol/l). Metformin, on the other hand, had no effect on the ncNOS activity at a basal level of 7 mmol/l glucose. However, addition of metformin totally suppressed the glibenclamide-induced iNOS activity and, moreover, reduced ncNOS activity back to control level, an event being reflected in a marked augmentation of glibenclamide-stimulated insulin release. Notably, it should be recalled that we have shown previously that N^G^-nitro-L-arginine methylester (L-NAME) a selective ncNOS inhibitor in isolated murine islets [17,27] brought about a highly significant increase in glibenclamide-induced insulin release both *in vitro* and *in vivo* [28]. Importantly, after intravenous pretreatment of mice with L-NAME, 5 sec prior to an intravenous injection of a half-maximal dose of glibenclamide, the acute insulin release was more than 2-fold higher than after glibenclamide alone, thus indicating a marked restraining action of sulfonylurea-induced ncNOS activity on the insulin-releasing process. L-NAME had no effect on basal insulin release [28]. These results thus showed that glibenclamide similar to glucose [6,7,17,19], has a dual action on the release process through markedly reducing its own stimulatory action by a negative feedback effect exerted by a simultaneous activation of ncNOS-derived NO. Note that the short-term effect by metformin on augmenting insulin release is more pronounced after glibenclamide than after glucose and thus correlated to metformin´s strongly suppressive effect on ncNOS activity in the glibenclamide experiments.

With regard to the effects of high glucose levels on the NOS-NO system, upregulation of islet iNOS-derived NO over time is suggested to contribute to β-cell dysfunction during development and progression to manifest nonimmunogenic type 2 diabetes both in rat and man [9,18,19]. Notably, it has been shown that transgenic mice overexpressing iNOS in their β-cells develop severe diabetes and islet destruction without any signs of insulitis [29]. From our present culture experiments with glibenclamide or high glucose with a confocal image of high iNOS protein expression and fluorescence intensity as well as a subsequent reduction of the insulin secretory capacity following a glucose challenge, it seems conceivable to assume that β-cell failure after long term hyperglycemia as well as after long term monotherapy with glibenclamide is, at a considerable part, due to the induction of iNOS and a long-time overproduction of iNOS-derived NO in the beta-cells. Furthermore, our findings that metformin in both short- and long-time experiments almost abolished iNOS activity both in combination with glibenclamide and high glucose lend support to our current hypothesis, that overactivity of the beta-cell NOS-NO system contributes significantly to the development of nonimmunogenic type 2 diabetes. Such a hypothesis is underscored by our previous finding showing that young Goto-Kakizaki (GK) rats, an animal model of nonobese type 2 diabetes, display both an increased ncNOS activity and excessive levels of iNOS-derived NO in their islets coincident with impaired glucose-stimulated insulin release [18,19]. Moreover, nonobese human type 2 diabetics show a marked upregulation of islet iNOS expression and activity coincident with beta-cell dysfunction [9]. In fact, a previous observation strongly supporting our hypothesis, that overactivity of the islet NOS-NO system might be a key inducer of beta-cell dysfunction, is our finding of a marked down-regulation of this system in islets from the obese *ob/ob* mice [29]. Thus freshly isolated islets from both young and adult hyperglycemic *ob/ob* mice are almost devoid of iNOS activity and display markedly reduced ncNOS activity coincident with a high rate of insulin release and the absence of a beta-cell demise in spite of their longstanding obesity and hyperglycemia [30]. Furthermore, it was recently shown that islets from obese human individuals as well as from obese Zucker fa/fa rats displayed a drastic increase in catalytically inactive ncNOS enzyme protein coincident with an augmented glucose-stimulated insulin release [12]. These data thus strengthen our hypothesis of ncNOS-derived NO as an important negative modulator of glucose-stimulated insulin release.

Since the pattern of ncNOS and iNOS activities found in the presence of glibenclamide at 7 mmol/l glucose is almost identical to the pattern found at high glucose (20 mmol/l), it seems conceivable to conclude that glibenclamide, and most probably also other sulfonylureas, has the ability to induce a previously less understood beta-cell dysfunction through imposing overactivity of the NOS-NO system in the background of its well-known stimulation of insulin release through closure of the beta-cell K_ATP_ channels and subsequent increase in [Ca^2+^]_i_ as well as the recently described stimulation of Epac2A [31]. Thus glibenclamide apparently has a double-edged effect by stimulating insulin release and at the same time inducing a significant restraining effect on secretion by imposing overactivity of the NOS-NO system of the beta-cell.

It should be recalled that strongly increased levels of islet NO production in both rodent and human islets have been reported to have an inhibitory influence on both nonoxidative and oxidative metabolism of glucose leading to a reduced ATP/ADP ratio and a reduced insulin secretion [14,32]. Excessive NO production is eventually followed by damaging levels of RNS and ROS [33]. However, it should be stressed upon that the intimate molecular mechanisms of NO action within the beta-cell, both in physiology and pathophysiology, are still unrevealed and future attention should possibly be directed to the influence of nitrosylation, denitrosylation and tyrosine nitration mechanisms along the insulin secretory signalling pathways [33–37].

The mechanisms underlying the beneficial effects of metformin on the beta-cell have for long been unclear. Metformin has been suggested to be a positive modulator of glucose-stimulated insulin release through activation of AMP-activated protein kinase (AMPK), but recent studies concluded that AMPK in fact is a negative modulator of the release process [38]. Previous [3,4] and recent [39] data from experiments with human islets have shown that when culturing the islets at high glucose, co-culturing with metformin has been able to counteract and restore the reduced ATP/ADP ratio, the increased activity of mitochondrial complex I, and the reduced insulin secretion induced by the glucotoxicity. Moreover, glucotoxic damage to beta-cell mitochondria seen at the ultrastructural level seemed to be restored by metformin [39]. Hence, an excessive NO production within the beta-cell as found in the present study following the glibenclamide treatment as well as after persistently elevated glucose levels might well explain most of these negative effects, which thus, at least in part, can be ameliorated by metformin.

As previously mentioned, metformin is recommended as a first-line agent in the treatment of impaired glucose tolerance and type 2 diabetes mainly because of its ability to reduce hepatic glucose production and increase insulin-stimulated glucose uptake in muscle and fat [1,2,5]. The present experimental data now speak in favor of also including a beneficial action of metformin on the β-cell by preserving the insulin secretory function and beta-cell viability through suppressing an excessive activity of the islet NOS-NO system during exposure to glibenclamide or high glucose. Moreover, recently metformin has also been shown to acutely increase the plasma levels of GLP-1 [40,41]. In addition it was found that metformin increased the expression of both GLP-1 and GIP receptors in the beta-cells by a PPARα-dependent mechanism [40,41]. Further it was recently reported [42] that a delayed-release formulation of metformin exhibited an unexpected lowering of plasma glucose in healthy humans in the face of very low plasma levels of the drug, pointing to a predominant effect of such a pharmaceutical formulation of metformin being exerted on the lower bowel, presumably on the GLP-1 producing cells. All these findings speak in favor of metformin and/or chemical and pharmaceutical variants thereof, being active both in bowel and in plasma, in suitable cases could substitute for the expensive incretin-based therapy, the long-time safety of which is currently somewhat questionable [43,44]. Moreover, the long-time and ultimate effects of such therapy on endogenous GLP-1 secretion are unknown, and with regard to DPP-4 inhibitors, negative effects on the cardiovascular system by raised levels of GIP [45] have recently been reported. In contrast, metformin has been shown to lower cardiovascular morbidity and even to display anticancer properties [2]. It is also noteworthy that metformin can increase the concentration of bile acids in the intestines, which in turn can stimulate GLP-1 secretion [46]. Interestingly as previously shown [9,47], NO seems not to negatively influence the positive modulation of insulin secretion induced by the cyclic AMP system. Thus therapeutic addition of certain phosphodiesterase (PDE) inhibitors [9] might also be useful in the future especially since type 2 diabetics display highly increased expression levels of PDE3 in their islets [9].

From the present data we conclude that the islet NOS-NO system is a more important regulator of insulin secretory mechanisms than previously anticipated. Imposed and persisting overstimulation of this system by glibenclamide and/or high glucose will result in an excessive production of NO with the capability of inhibiting crucial steps along the insulin secretory signalling pathways and in the long term eventually lead to a reduced beta-cell viability. The negative influence of the concealed dysfunctional NO effects induced by glibenclamide or high glucose on beta-cell activity can be counteracted, relieved and to a significant part explained by the ability of metformin to downregulate this imposed overactivity of the NOS-NO system both in short- and long-time perspective. Hence, in this new perspective, the beta-cell failure after long term sulfonylurea treatment is now more understandable and the present experimental results, from the point of view of the beta-cell, strongly suggest sulfonylurea monotherapy in type 2 diabetes being avoided.
